# Circadian protein BMAL1 promotes breast cancer cell invasion and metastasis by up-regulating matrix metalloproteinase9 expression

**DOI:** 10.1186/s12935-019-0902-2

**Published:** 2019-07-16

**Authors:** Jian Wang, Shujing Li, Xiahui Li, Bowen Li, Yanan Li, Kangkai Xia, Yuxi Yang, Sattout Aman, Miao Wang, Huijian Wu

**Affiliations:** Province Key Laboratory of Protein Modification and Occurrence of Disease, Dalian University of Technology, 2 Ling Gong Road, Dalian, 116024 Liaoning Province China

**Keywords:** BMAL1, MMP9, NF-κB, Invasion, Metastasis

## Abstract

**Background:**

Metastasis is an important factor in the poor prognosis of breast cancer. As an important core clock protein, brain and muscle arnt-like 1 (BMAL1) is closely related to tumorigenesis. However, the molecular mechanisms that mediate the role of BMAL1 in invasion and metastasis remain largely unknown. In this study, we investigated the BMAL1 may take a crucial effect in the progression of breast cancer cells.

**Methods:**

BMAL1 and MMP9 expression was measured in breast cell lines. Transwell and scratch wound-healing assays were used to detect the movement of cells and MTT assays and clonal formation assays were used to assess cells’ proliferation. The effects of BMAL1 on the MMP9/NF-κB pathway were examined by western blotting, co-immunoprecipitation and mammalian two-hybrid.

**Results:**

In our study, it showed that cell migration and invasion were significantly enhanced when overexpressed BMAL1. Functionally, overexpression BMAL1 significantly increased the mRNA and protein level of matrix metalloproteinase9 (MMP9) and improved the activity of MMP9. Moreover, BMAL1 activated the NF-κB signaling pathway by increasing the phosphorylation of IκB and promoted human MMP9 promoter activity by interacting with NF-kB p65, leading to increased expression of MMP9. When overexpressed BMAL1, CBP (CREB binding protein) was recruited to enhance the activity of p65 and further activate the NF-κB signaling pathway to regulate the expression of its downstream target genes, including MMP9, TNFα, uPA and IL8, and then promote the invasion and metastasis of breast cancer cells.

**Conclusions:**

This study confirmed a new mechanism by which BMAL1 up-regulated MMP9 expression to increase breast cancer metastasis, to provide research support for the prevention and treatment of breast cancer.

## Background

There are obvious circadian rhythmic changes in the process of maintaining homeostasis and adapting to the external environment [1]. The circadian rhythm refers to an approximately 24 h biological rhythm produced by the endogenous circadian oscillator [2]. The central pacemaker of this rhythm is located in the suprachiasmatic nucleus (SCN) of the hypothalamus. The positive and negative feedback loops of the circadian proteins BMAL1, CLOCK, PER1, and PER2 regulate various physiological processes of the human body, including blood pressure, hormone secretion, sleep and immune activities [3–6]. Circadian rhythm disorders may increase the risk of diseases such as cardiovascular disease, immune system diseases and cancer [7–10].

As an important circadian protein, BMAL1 plays an important role in maintaining the normal and orderly life activities of organisms. In addition to controlling the function of biological rhythms, BMAL1 also plays important roles in aging, cardiovascular disease, immune diseases and cancer [11–14]. Current research revealed that BMAL1 is involved in the regulation of cell cycle and proliferation, suggesting that BMAL1 may take a crucial effect in tumorigenesis [15]. However, the mechanism of BMAL1 in the development of various cancers is not fully understood. For example, Jiang et al. [16] found that BMAL1 can induce apoptosis and G2/M phase arrest in a p53-dependent manner, thereby inhibiting the proliferation of colorectal cancer cells. And Elshazley et al. [17] found that *Bmal1* expression is increased in malignant pleural mesothelioma. Silencing BMAL1 leads to cell cycle disorder and increased apoptosis. BMAL1 is likely to play a role in cancer prevention in malignant pleural mesothelioma. These findings suggest that the mechanism by which BMAL1 regulates cancer is complex.

Breast cancer is the most common cancer in the world and has the highest cancer-related mortality rate among women. Although early diagnosis and treatment have made great progress, the invention rate of breast cancer is still on the rise [18]. Tumor metastasis is an important factor in the poor prognosis of the tumor. The metastasis is firstly local invasion, intravascular infiltration and subsequent transmission through the circulatory system and lymphatic system. Next, the disseminated tumor cells infiltrate into the parenchyma of the distal organ, eventually leading to the formation of micrometastases, in turn, forms secondary tumors [19].

Tumor metastasis involves the participation of many molecules, As a result, the adhesion of cancer cells is reduced, and the detached cancer cells are connected with the basement membrane or the extracellular matrix (ECM). Among which MMP9 has been shown to be involved in tumor invasion and metastasis, and its activity is significantly enhanced during tumor progression. MMP9 is an important member of the ECM metalloproteinase family. Also, MMP9 is involved in the degradation process of the tumor extracellular matrix and is a mediating factor for local invasion and distant metastasis of tumor [20]. Studies have shown that MMP9 can damage the integrity of the extracellular matrix, which opens an important channel for the invasion of tumor cells and accelerates the formation of tumor deterioration. The expression of MMP9 decided by the transcription and its regulation of transcriptional activity focused on the transcription start site 670 bp upstream of nucleotide sequences, including the AP-l, NF-κB, Spl and Ets-l binding sites [13]. MMP9 is mainly expressed in glandular epithelial tissue of the breast, so it is of great importance for studying the expression of MMP9 in breast cancer [21].

The nuclear factor of B (NF-κB) family mainly consists of five members: p65 (Rel A), Rel B, c-rel, NF-κB1(p105/p50) and NF-κB2(p100/p52). The classical regulation mechanism of NF-κB is: at rest, IκBα and p65/p50, forms a complex, which is inactive in the cytoplasm. When cells are stimulated by extracellular signals, IκBα is phosphorylated and degraded at the same time, and dissociated from p65 and p50, exposing NF-κB (i.e. p65 and p50) to nuclear localization sites. Free p65 and p50 are rapidly transferred to the nucleus and bind to specific sequences to regulate the transcription of a variety of genes related to immunity and inflammation, so as to regulate cell growth, autoimmunity and inflammation [22, 23].

In this study, we demonstrated that BMAL1 can promote the invasion and metastasis of breast cancer cells, and at least partially up-regulate the expression of MMP9. Mechanistically, BMAL1 can promote the expression of MMP9 at the transcription level, which is partly dependent on the association with NF-κB p65. The interaction was accompanied by the recruitment of CBP that served to promote the acetylation level of p65. The expressions of BMAL1 and MMP9 were also examined in different human breast cancer cell lines, showing that the expression of BMAL1 was higher in more invasive breast cancer cells and was positively correlated with the expression of MMP9.

## Methods

### Cell culture and transfection

HEK-293T, MCF-7, T47D, ZR-75-30 and MDA-MB-231 cells have been used in our previous study and were cultured as previously described [23, 24]. Cells were grown at 37 °C in a humidified 5% CO_2_ atmosphere and then the cells were transfected with the appropriate plasmids using Lipofectamine 2000 (Invitrogen, Auckland, New Zealand). Rabbit anti-BMAL1, anti-p50 and anti-p65 antibodies were purchased from Abcam (Abcam, Cambridge, MA, USA). Rabbit anti-Flag, anti-GFP, anti-HA, anti-Myc, mouse anti-Flag antibodies were purchased from Sigma (Sigma, Saint Louis, MO, USA). Mouse anti-MMP9 antibody was purchased from Santa Cruz (Santa Cruz, Dallas, CA, USA).

### Plasmid construction

Human Bmal1 was cloned from a human cDNA library using the forward primer Bmal1-F: 5′-cggaattcccaccgactaccaggaaagt-3′, and Bmal1-R: 5′-ccgctcgagcgctaaagtcaacgggacca-3′, and the amplified *Bmal1* DNA fragment was inserted into the expression vector pcDNA3.1-Flag and pcDNA3.1-HA at the EcoRI-XhoI sites. Human p50 was amplified from a human cDNA library using the following primers p50-F: 5′-ccggaattccgcagaagatgatcca-3′ and p50-R: 5′-ccctcgaggtcatagaaagaggttatcc-3′, and the amplified p50 DNA fragment was inserted into the expression vector pcDNA3.1-3×Flag at the EcoRI-XhoI sites. Human p65 was amplified from a human cDNA library using the following primers p65-F: 5′-ccgctcgagggatggacgaactgttcc-3′ and p65-R 5′-ccgggtaccggagctgatctgactc-3′, and the amplified p65 DNA fragment was inserted into the expression vector pEGFP-N3 at the XhoI-KpnI sites. pcDNA3.1-Flag, Myc-CBP and pGL3 vector and the pNF-κB luciferase reporter construct were acquired as previously described [21].

### Transwell assays

ZR-75-30 or MDA-MB-231 cells were transfected with appropriate plasmids. Transwell assays were performed as previously described [24]. After 24 h transfection, the cells were suspended in serum-free medium and counted. The suspension containing 1 × 10^5^ cells was inoculated in the upper chamber with (invasion) or without (migration) Matrigel and after 24 or 48 h, the cells transferred to the lower chamber were stained and then photographed.

### Scratch wound-healing assays

Scratch wound-healing were performed as previously described [24].

### Cell growth assays

MTT assays and Clonal formation assays were used to assess cells’ proliferation. T47D or ZR-75-30 cells were transfected with appropriate plasmids, after 24 h transfected, then seeded in 96-well plates, with 1000 or 2000 cells in each well and then subjected to MTT assay performed with a commercial kit (KeyGen) according to the manufacturer’s protocol. The absorbance of the samples was read at 490 nm. For clonal formation assays, ZR-75-30 or MDA-MB-231 cells transfected with the respective plasmids were immobilized with ethanol and stained with 0.1% crystal violet and then photographed.

### Luciferase reporter assay

Promoter activity was examined by a luciferase assay system. ZR-75-30 cells were inoculated in 24-well plates, and cultured for 24 h. Then the cells were transfected with corresponding plasmids using Lipofectamine 2000 according to the company’s specification. Twenty-four hours after transfection, the cells were subjected to luciferase and Renilla activity assays according to the manufacturer’s instructions (Promega, Madison, WI, USA).

### Construction and mutagenesis of the human MMP9 promoter-reporter

Studies have shown that BMAL1 is involved in transcription initiation [25]. Moreover,response elements needed to regulate transcription were found at 670 bp near the MMP9 promoter [26]. Therefore, we replicated about 800 bp of MMP9 TSS into the pGl3-Luc luciferase reporter vector and obtained luciferase plasmids. An MMP9 promoter fragment spanning nucleotides 795 to + 19 was synthesized from human genomic DNA (Promega) from ZR-75-30 cells by PCR using the forward primer 5′-ggggtacctttagggacaaagagcccc-3′ and reverse primer 5′-ccgctcgaggtgagggcagaggtgtc-3′. The amplified PCR product was inserted into the pGL3 vector (Promega) at the KpnI and XhoI sites, yielding pMMP9-Luc (795, + 19). A series of truncated human MMP9 promoter fragments were synthesized by PCR using the pMMP9-Luc (795, + 19) plasmid as template. Forward primer sequence used in this construction was 5′-ggggtacccctagcagagcccattcct-3′ (− 587 to + 19), One reverse primer, + 19R, was used to generate construct. Site-specific mutations of the NF-kB-binding site within the MMP9 promoter were performed with the QuickChange site-directed mutagenesis system (Stratagene) using pMMP9-Luc (795, + 19) as a template. Primer sequences used to generate point mutation was 5′-ttgccccagtggaattggccagccttgcctagc-3′ for disruption of the NF-κB binding site.

### Western blot, CoIP assays

Western blot and co-immunoprecipitation (CoIP) assays were conducted as previously described [27].

### Mammalian two hybrid assay

The checkmate TM mammalian two-hybrid system was obtained from Promega. BMAL1 and p65 were subcloned into BamHI–SalI cut pACT and pBIND, respectively.

### RNA extraction and RT-PCR

Total RNA was isolated from ZR-75-30 cells using RNAiso Reagent (Takara, Dalian, China). Total RNA (3 μg) was reverse transcribed using oligo (dT) primer and a Reverse Transcription System (Takara). The single-stranded cDNA was amplified by PCR using specific primers: MMP9: 5′-tactgtgcctttgagtccg-3′ (forward) and 5′-ttgtcggcgataaggaag-3′ (reverse); Bmal1: 5′-ccaccgactaccaggaaagt-3′ (forward) and 5′-cgctaaagtcaacgggacca-3′ (reverse); and GAPDH: 5′-gggttgaaccatgagaagt-3′ (forward) and 5′-gactgtggtcatgagtcct-3′ (reverse). The PCR products were analyzed by 1% agarose gel electrophoresis.

### Real-time PCR

Appropriate plasmids were transfected into ZR-75-30 cells and 24 h, the total cDNA was synthesized as described above. Relative mRNA levels were determined using the ABI Prism 7500 sequence detection system with SYBR Premix Ex Taq (Takara) as previously described [28]. Expression of target genes was determined according to the 2−∆∆CT method using GAPDH as a reference gene. The following primer sequences were used: MMP9, 5′-tactgtgcctttgagtccg-3′ (forward) and 5′-ttgtcggcgataaggaag-3′ (reverse); Bmal1, 5′-tgcaagggaagctcacagtc-3′ (forward) and 5′-gattggtggcacctcttaatg-3′ (reverse); TNF-a, 5′-cccaggcagtcagatcatcttc-3′ (forward) and 5′-agctgcccctcagcttga-3′ (reverse); IL8, 5′-ttttgccaaggagtgctaaaga-3′ (forward) and 5′-aaccctctgcacccagttttc-3′ (reverse); VEGFA, 5′-cgggaaccagatctctcacc-3′ (forward) and 5′-aaaatggcgaatccaattcc-3′ (reverse) and uPA, 5′-agtgtcagcagccccact-3′ (forward) and 5′-ccccctgagtctccctgg-3′ (reverse).

### Gelatin zymography

According to MMP Zymography Assay Kit(XF-P17750, ZR-75-30 cells were transfected with appropriate plasmids. After 24 h transfection, the cells were removed from the culture medium, and the serum-free medium was added for further 24 h culture. Culture supernatants were collected and run on a gelatin containing 8% SDS-PAGE gel. The gel was treated in the Buffer A followed by incubation in Buffer B at 37 °C for overnight. Then the gel was stained and destained. MMP9 bands can be seen at the 92KD position.

### Immunofluorescence

Cells were seeded on the cover glass and transfected with HA-BMAL1 and GFP-p65 plasmids. After 1 day, cells were washed with PBS and fixed with 1% paraformaldehyde at room temperature for 15 min, then followed by permeabilized with methanol at − 20 °C for 40 min, and blocked with 0.8% BSA for 1 h. After that, the cells were incubated with the appropriate antibody for overnight then rinsed with PBS and incubated with secondary antibody for 1 h. The cover slips were mounted on glass slides with mounting medium containing 4′,6-diamidino-2-phenylindole (DAPI), and examined and photographed using a Nikon TE2000-U microscope.

### Statistical analysis

Data were examined as mean ± SDs from at least three independent experiments. Un-paired t-test was used when the results from the two groups were compared. Statistical analyses were carried out by one-way analysis of variance with Bonferroni’s multiple-comparison correction for comparison among three or more groups. Statistical significance was considered at the p < 0.05 level.

## Result

### BMAL1 regulates breast cancer cells migration and invasion

**Fig. 1:**
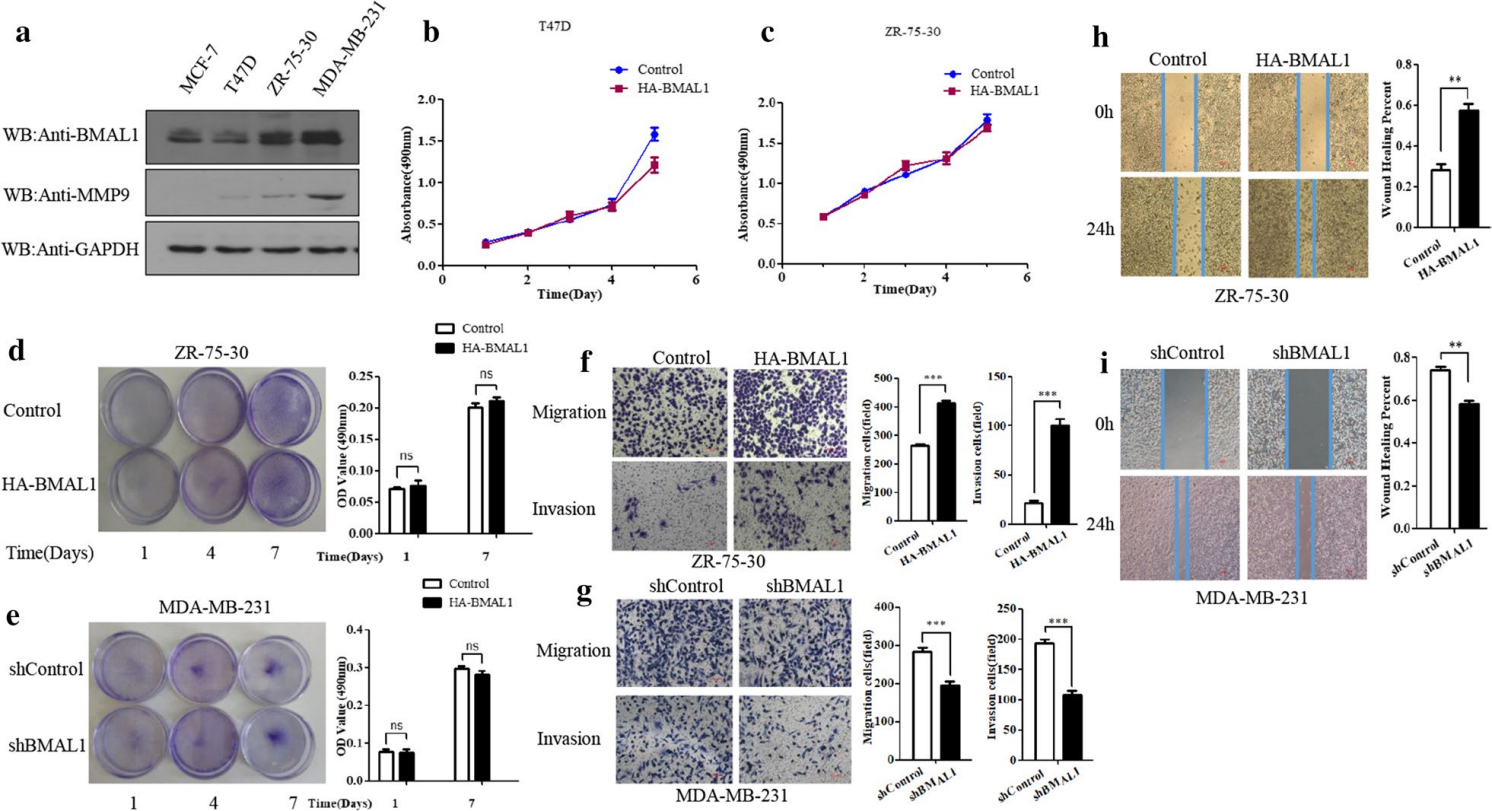
The effect of BMAL1 on the proliferation and cellular motility of breast cancer cells. **a** Western blot analysis of BMAL1 and MMP9 expression in MCF-7, T47D, ZR-75-30, and MDA-MB-231 cell lines. Experiments were repeated at least three times. **b**, **c** MTT assays assessing the effects of BMAL1 overexpression on the proliferation of ZR-75-30 and T47D cells. **d** Clonal formation assays assessing the effects of BMAL1 overexpression on the proliferation of ZR-75-30 cells. **e** Clonal formation assays assessing the effects of BMAL1 knockdown on the proliferation of MDA-MB-231 cells. **f**, **g** Transwell migration and invasion assays assessing the effects of BMAL1 overexpression or knockdown on the motility of ZR-75-30 or MDA-MB-231 cells. Cell migration and invasion assays were performed in 24-well chambers without or with Matrigel. Cells (1 × 10^5^ per well) were transfected with HA-BMAL1 and control vector and shBMAL1 or shControl then plated in the upper chamber. After 24 h of incubation, the migrating and invading cells on the lower surface of the filter were stained and counted (left). The bar graphs show the number of migrating and invading cells for each category of cells repeated three times (right). **h**, **i** Scratch wound-healing assay evaluating the effects of BMAL1 overexpression or knockdown on the cellular motility and invasion ability of ZR-75-30 or MDA-MB-231 cells. Data are presented as mean ± SD (p < 0.05, significant; ns, not significant; *, p < 0.05; **, p < 0.01; ***, p < 0.001)

Previous studies have shown that deficiency of circadian proteins in breast epithelial cells can increase the susceptibility of breast cancer and lead to more aggressive breast cancer. However, recent studies have shown that circadian protein BMAL1 has a cancer-promoting effect in breast cancer [14], but the specific molecular mechanism is not well understood. In order to explore the role of BMAL1 in the occurrence and development of breast cancer, therefore, we selected breast cancer cell lines containing MCF7, T47D, MDA-MB-231 and ZR-75-30 cells. Almost no endogenous MMP9 was detected in MCF7 cells using Western blot analysis. Relatively higher expression of BMAL1 was identified in MDA-MB-231 cells than in T47D and ZR-75-30 cells, consistent with the expression of MMP9 in breast cancer cells (Fig. 1a). At the same time, we found that BMAL1 expression is higher in invasive cancer cells than in non-invasive cancer cells. Then, we studied the effect of BMAL1 on the proliferation and invasion ability of breast cancer by overexpressing BMAL1 through MTT, clonal formation, Scratch wound healing and Transwell migration assays. MTT and clonal formation assays showed that overexpression or knockdown of BMAL1 had no significant effect on the proliferation of ZR-75-30, T47D or MDA-MB-231 cells (Fig. 1b–e). The results of Scratch wound healing and Transwell migration assays showed that overexpressing of BMAL1 could promote the invasion and metastasis of ZR-75-30 cells (Fig. 1f, h). In contrast, knockdown of endogenous BMAL1 in MDA-MB-231 cells resulted in the significant down-regulation of cells’ invasion and metastasis (Fig. 1g, i). These above results indicate that BMAL1 can promote the invasion and metastasis of breast cancer cells.

### BMAL1 regulates MMP9 mRNA and protein levels and promoter activity

**Fig. 2:**
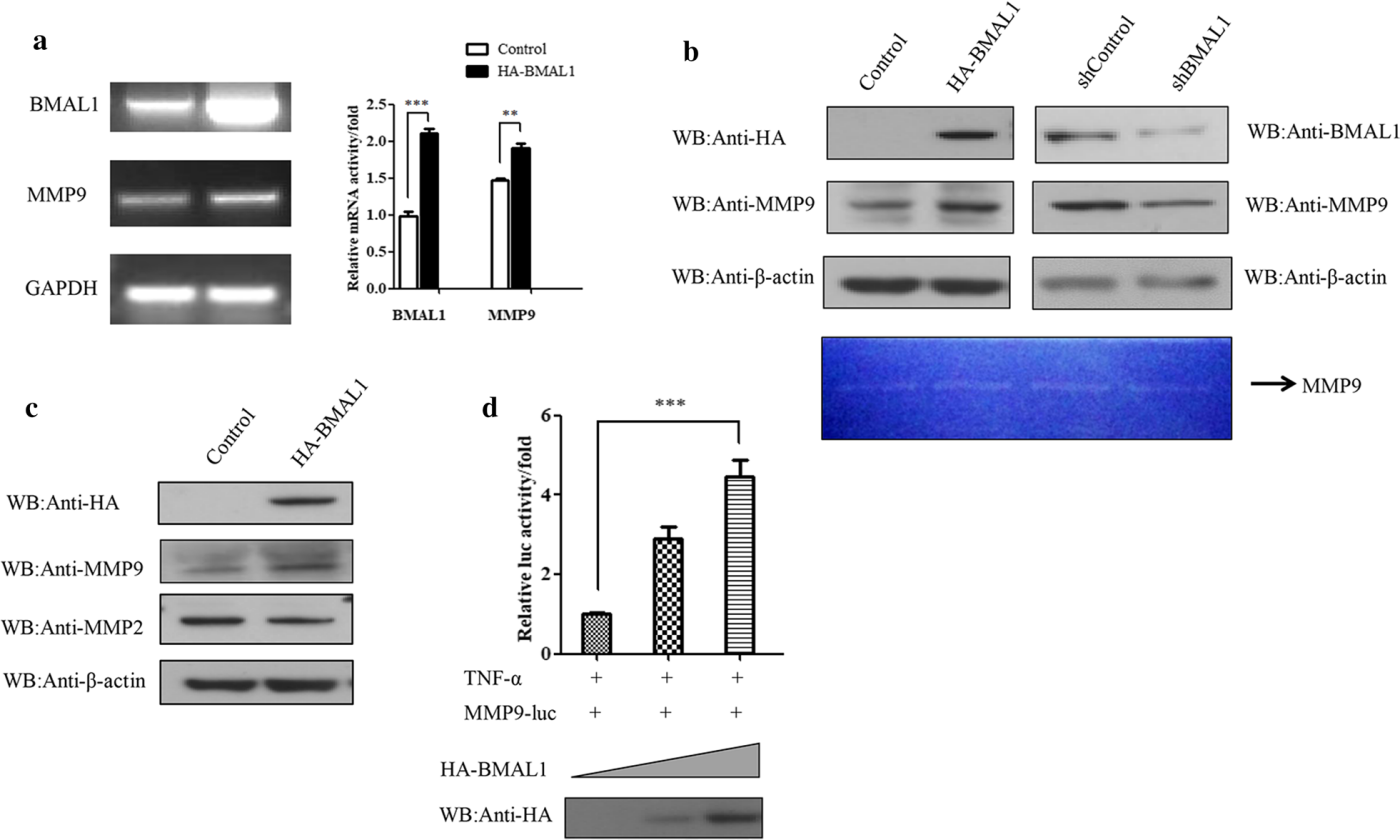
The role of BMAL1 on MMP9 mRNA and protein levels along with the promoter activity of MMP9. **a** RT-PCR showing the effects of BMAL1 overexpression on the mRNA levels of MMP9 in ZR-75-30 cells. The bar graphs show the fold changes in relative mRNA levels normalized against GAPDH (right). **b**, **c** T47D and ZR-75-30 cells were transfected with HA-BMAL1 or control vector, and shBMAL1 or shControl. The samples (cells and medium) were subjected to western blot analysis with the indicated antibodies and zymography. **d** ZR-75-30 cells were transfected with pMMP9-luciferase and HA-BMAL1. Luciferase activity was measured and normalized against the b-galactosidase activity. For comparison, the pMMP9-Luc activity level of control cells was set to 1. All experiments were repeated at least three times. Data are presented as mean ± SD (p < 0.05, significant; **, p < 0.01; ***, p < 0.001)

Invasion and metastasis are a series of dynamic processes involving multiple steps and genes in malignant tumors. Among of them, matrix metalloproteinase 2 (MMP2) and matrix metalloproteinase 9 (MMP9) due to degradation of ECM IV type collagen, the primary components of the basement membrane damaged prompted local invasion and distant metastasis and have the closest relations with invasion and metastasis of tumor [29, 30], real-time quantitative PCR assay and Western blot assay were used to detect the changes of MMP2 and MMP9 when overexpressed BMAL1. Indeed, overexpression of BMAL1 in T47D and ZR-75-30 cells increased MMP9 mRNA level (Fig. 2a). Western blot result revealed an increased level of MMP9 protein upon BMAL1 overexpression, consistent with the changes observed at the mRNA level. More importantly, the activity of MMP9 was also determined by gelatinase assay. As expected, MMP9 activity was increased with BMAL1 overexpression and decreased with BMAL1 knockout (Fig. 2b), but the protein level of MMP2 was not affected (Fig. 2c).

The regulation of MMP9 mRNA level by BMAL1 was further studied using luciferase reporter assay. ZR-75-30 cells transfected with increasing doses of BMAL1 induced a dose-dependent promotion of MMP9-driven luciferase activity (Fig. 2d). In a word, the results above showed that BMAL1 could promote the expression of MMP9 protein by transcriptionally activating the activity of the MMP9 gene promoter.

### BMAL1 regulates MMP9 expression partly by the NF-κB signaling pathway

**Fig. 3:**
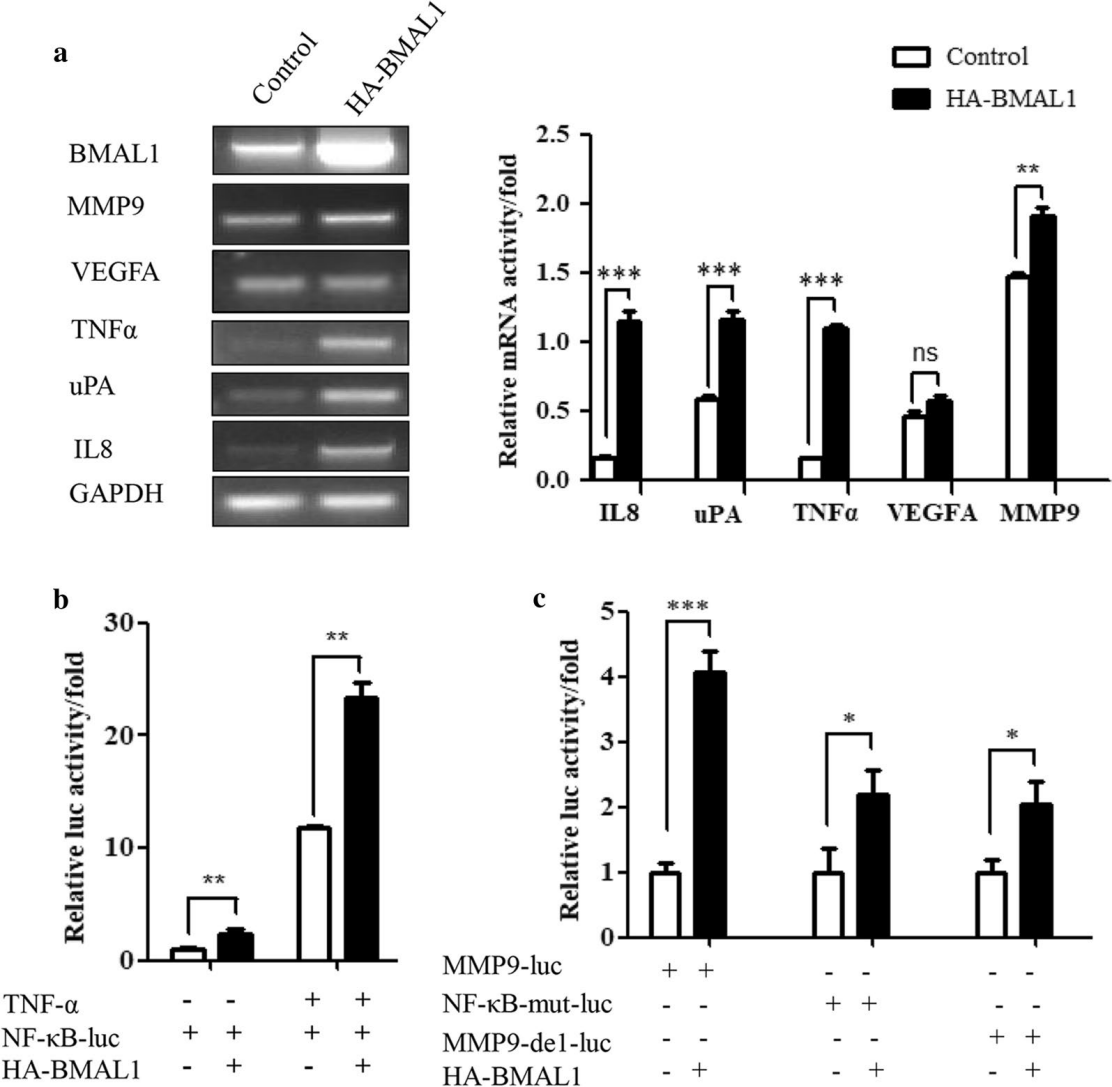
The role of BMAL1 on NF-κB signaling pathway. **a** Real-time PCR showing the effects of BMAL1 overexpression on the mRNA expression levels of TNF-a, VEGFA, and uPA and IL8 in ZR-75-30 cells. All experiments were repeated at least three times. **b** NF-κB promoter reporter plasmid, and HA-BMAL1 or control vector were co-transfected into ZR-75-30 cells to determine luciferase activities. **c** Site-specific mutants or deletion for NF-kB (MMP9-del or NF-kB-mut), derived from the − 795/+ 19 construct of human MMP9 promoter reporter plasmids, and HA-BMAL1 or control vector were co-transfected into ZR-75-30 cells to determine luciferase activities. All experiments were repeated at least three times. Data are presented as mean ± SD (p < 0.05, significant; ns, not significant; *, p < 0.05; **, p < 0.01; ***, p < 0.001)

As we all know, MMP9 is the potential target of NF-κB, therefore, we want to detect whether the regulation of MMP9 is achieved by regulating the NF-κB signaling pathway. We first examined the effect of BMAL1 on the NF-κB luciferase reporter gene and found that BMAL1 could activate the activity of the NF-κB reporter gene (Fig. 3b), and the expression of NF-κB downstream target genes, including MMP9, TNFα, uPA and IL8 significantly increased upon BMAL1 overexpression (Fig. 3a). Then we constructed two different MMP9 reporter genes, and the deletion of the NF-κB site or the mutation of the NF-κB site further verified whether the effect of BMAL1 on MMP9 was achieved through the NF-κB site. It was found that the effect of BMAL1 on the MMP9 reporter gene was significantly reduced after the deletion or mutation of the NF-κB site (Fig. 3c). The above experimental results proved that the effect of BMAL1 on the MMP9 promoter was indeed achieved partly by the NF-κB signaling pathway.

### BMAL1 interacts with p65 in breast cancer cells

**Fig. 4:**
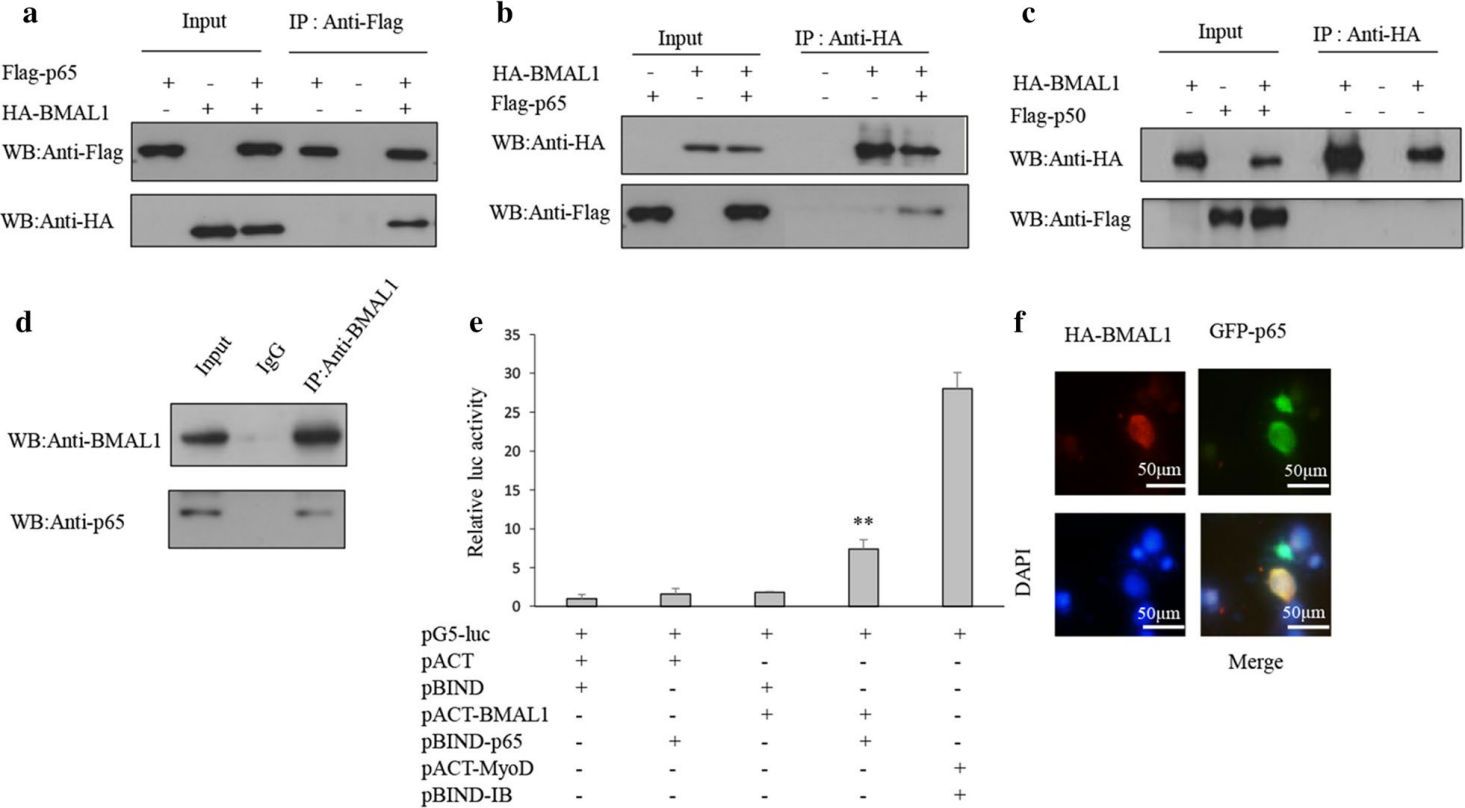
The interaction between BMAL1 and p65. **a**, **b** HEK 293T cells transfected with Flag-p65 only, HA-BMAL1 only or with Flag-p65 and HA-BMAL1 were subjected to immunoprecipitation with anti-Flag antibody followed by Western blot with anti-HA antibody or vice versa. **c** HEK 293T cells transfected with HA-BMAL1 only, Flag-p50 only or with HA-BMAL1 and Flag-p50 were subjected to immunoprecipitation with anti-HA antibody followed by Western blot with anti-Flag antibody or vice versa. **d** ZR-75-30 cells were subjected to immunoprecipitation with anti-BMAL1 antibody followed by Western blot with anti-p65 antibodies. IgG was used as a negative control. **e** Direct interaction between BMAL1 and p65 as detected using a mammalian two-hybrid system. BMAL1 and p65 were expressed from pACT-BMAL1 and pBIND-p65, respectively, whereas the empty vectors pACT and p-BIND were expressed as controls, as indicated with the pG5-luc reporter in COS-7 cells. Cells were transfected with pBIND-ID and pACT-MyoD as a positive control. Luciferase activity was measured after 24 h of transfection. **p < 0.01 compared with cells transfected with pACT and pBIND. **f** Localization of Bmal1 and p65 in MCF-7. MCF-7 cells transfected with GFP-p65 and HA-BMAL1 were stained with rabbit anti-HA antibody and tetramethylrhodamine isothiocyanate (TRITC)-conjugated anti-rabbit IgG. GFP-p65 appeared as a green signal when visualized by fluorescence microscopy. Nuclei were stained with 4,6-diamidino-2-phenylindole (DAPI). All experiments were repeated at least three times. Data are presented as means ± SD (p < 0.05, significant; *, p < 0.05; **, p < 0.01)

BMAL1 regulates the activity of MMP9 promoter by regulating the NF-kB signaling pathway, so we detected whether transcription factors NF-κB p50/p65 were involved in this process. Thus, CoIP experiments were performed in HEK 293T cells to examine the interaction between BMAL1 and p50/p65. The exogenous interaction of BMAL1 and p65 was identified in HEK 293T cells transfected with HA-BMAL1 and FLAG-p65 via CoIP assays, followed by using either anti-HA or anti-FLAG antibody (Fig. 4a, b). However, the interaction between exogenous BMAL1 and p50 were not detected in HEK 293T cells transfected with HA-BMAL1 and Flag-p50 using the same CoIP assay (Fig. 4c). The endogenous association of BMAL1 and p65 was examined in ZR-75-30 cells (Fig. 4d). Moreover, the direct association of p65 and BMAL1 was also identified by the mammalian two-hybrid system (Fig. 4e). Meanwhile, immunofluorescence assay revealed the co-localization of GFP-p65 and HA-BMAL1 in the nucleus (Fig. 4f). In short, our results showed that BMAL1 could combine with p65 but not p50 and this association was important for the regulation of NF-κB element of the MMP9 promoter by BMAL1.

### BMAL1 doesn’t affect the binding of p65 to IκBα but recruits CBP

**Fig. 5:**
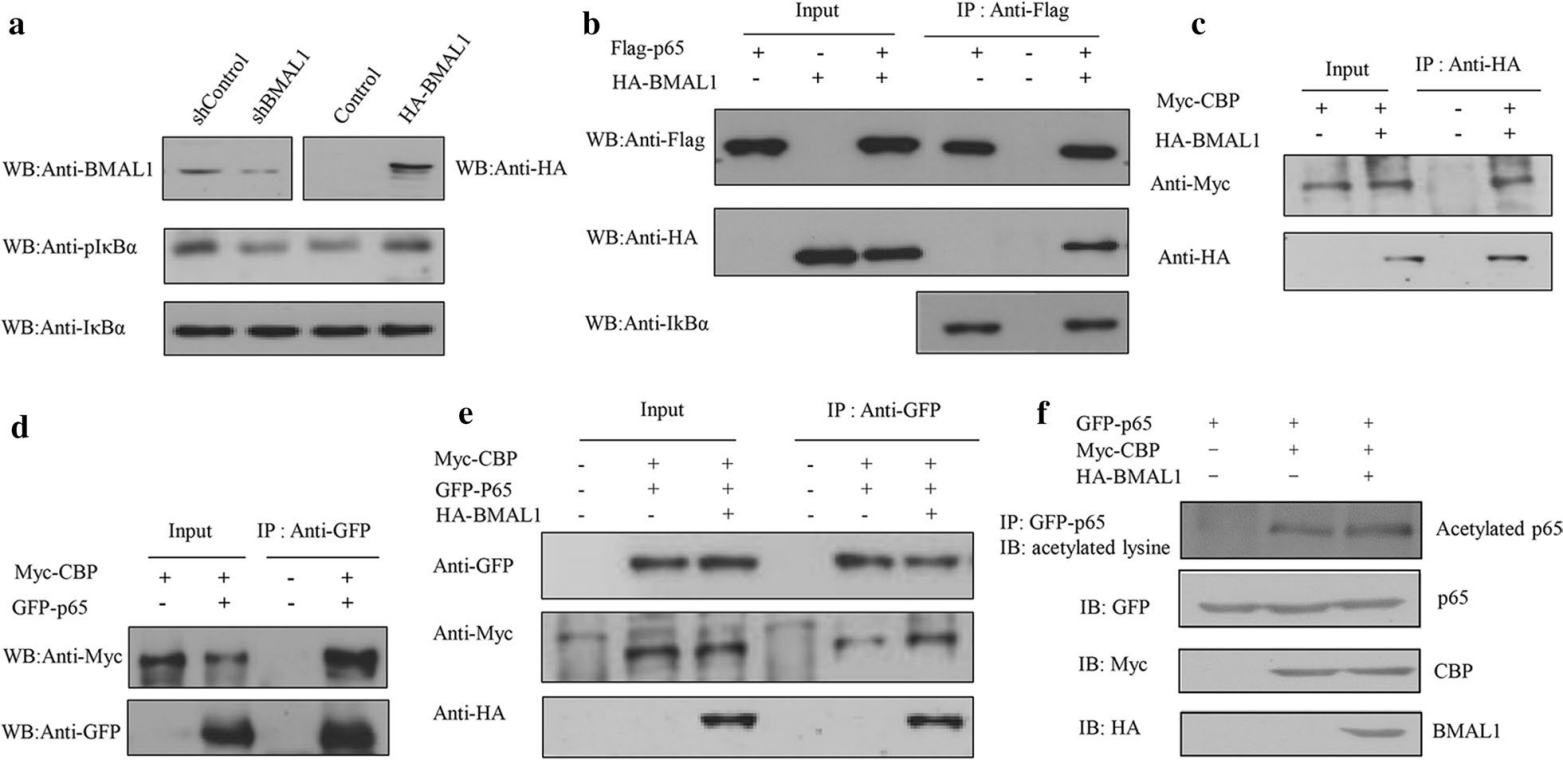
CBP is recruited by BMAL1 to transcriptionally regulate the acetylation of p65. **a** Western blot assay showed the effect of BMAL1 overexpression or knockdown on IκBα protein levels in ZR-75-30 cells. **b** The effect of the binding of p65 to IκBα was detected by immunoprecipitation with the indicated antibodies. **c** HEK 293T cells transfected with Myc-CBP only, or with HA-BMAL1 and Myc-CBP were subjected to immunoprecipitation with anti-Myc antibody followed by Western blot with anti-HA antibody or vice versa. **d** HEK 293T cells transfected with Myc-CBP only, or with GFP-p65 and Myc-CBP were subjected to immunoprecipitation with anti-Myc antibody followed by Western blot with anti-GFP antibody or vice versa. **e** HEK 293T cells transfected with control vector, GFP-p65 and Myc-CBP, or with GFP-p65, Myc-CBP and HA-BMAL1, were subjected to immunoprecipitation with anti-GFP antibody followed by Western blot with anti-HA and anti-Myc antibodies. **f** HEK 293T cells were transfected with the indicated plasmids for 24 h. The extract was immunoprecipitated with anti-GFP antibody and then probed with anti-acetylated lysine antibody. All experiments were repeated at least three times

At rest, IκBα and p65/p50 form a complex, which is inactive in the cytoplasm. When IκBα and p65 are separated, p65 can enter the nucleus to play a role. An essential step for NF-κB pathway activation is the phosphorylation of IκB. It showed that the phosphorylation level of IκBα was significantly increased with BMAL1 overexpression, conversely, knockdown of BMAL1 reduced the phosphorylation level of IκBα (Fig. 5a). However, CoIP assay showed that overexpression of BMAL1 did not affect the binding between p65 and IκBα in HEK 293T cells (Fig. 5b). Therefore, BMAL1 might play a role in the nucleus. Previous studies have demonstrated that the interaction between p65 and CBP could promote the acetylated level of p65 at several positions and this acetylation is directly related to the increased transcriptional activity of p65 [31]. Because there was an obvious effect of BMAL1 on the expression of p65, increasing of MMP9 expression by BMAL1 might also involve the recruitment of CBP. CoIP assays were performed to examine whether BMAL1 can interact with CBP, and the data revealed that BMAL1 specifically associated with CBP in HEK 293T cells transfected with HA-BMAL1 and Myc-CBP (Fig. 5c). Also, the interaction between CBP and p65 was identified in HEK 293T cells transfected with Myc-CBP and GFP-p65 by CoIP assay (Fig. 5d). Overexpression of BMAL1 markedly increasing the abundance of CBP in the CBP-p65 complex (Fig. 5e). Overexpression of BMAL1 together with CBP further promoted the acetylation level of p65 (Fig. 5f). Based on these findings, we illustrated that BMAL1 can up-regulate the transcriptional activity of p65 by promoting the interaction between CBP and p65 and the acetylation level of p65. Taken together, these results showed that BMAL1 could up-regulate the level of p65 protein and further affecting the NF-κB pathway.

## Discussion

In our study, we identified the promoting effect of BMAL1 on MMP9 transcription, which may provide a novel mechanism for human breast cancer invasion and metastasis. First, in breast cancer cells, BMAL1 promotes migration and invasion. Second, BMAL1 up-regulate MMP9 expression by promoting the transcription level of MMP9. Third, we found the underlying mechanism is that BMAL1 could recruit CBP, promoting the acetylation level of p65, further activating the expression of p65 and promoting the NF-κB signaling pathway. Fourth, In addition to MMP9, in the breast cancer cells, BMAL1 also up-regulated the expression of TNF-a, IL8, and uPA at the mRNA level, which are all target genes of NF-κB, suggesting a potential role of BMAL1 in NF-κB/MMP9 pathway. Finally, we demonstrated that BMAL1 could promote the invasiveness of breast cancer cells by up-regulating the expression and activity of MMP9. This study revealed the molecular mechanism by which BMAL1 regulates the invasion and metastasis of breast cancer cells through the inflammatory pathway NF-κB, and established the connection between the biological clock and the inflammatory response providing further theoretical support for the treatment and prevention of breast cancer.

Breast cancer is one of the major cancers threatening women’s life and health [18]. An epidemiological investigation revealed a phenomenon that women working rotational night shifts have an increased incidence of breast cancer [32], which suggested that the disorder of biological clock can affect the incidence and progression of breast cancer. Current studies suggest that circadian rhythm genes may affect tumor development by cell cycle regulation, metabolic changes, epithelial-mesenchymal transformation, proliferation and metastasis [33–36]. However, the mechanism of circadian rhythm gene in the development of breast cancer has not been fully understood. *Bmal1* is one of the core clock genes, which generates circadian rhythm through positive and negative feedback pathways, self-expression regulation and other auxiliary regulatory mechanisms [37]. Studies have found that BMAL1 may play an opposite role in different cancers, and it may regulate the proliferation of tumor cells through different pathways [16, 17]. Shanbeh et al. [38] found that BMAL1 has a close relationship with the risk of breast cancer. However, the exact mechanism of BMAL1’s effect on the risk of breast cancer needs to be further studied. Ma et al. [38] found that the CLOCK-BMAL1 complex controls the expression of the components of the RHOA-ROCK-CFL pathway, and then promotes cancer cell proliferation, migration, and invasion. In this study, it was found that overexpression of BMAL1 could promote the invasion and metastasis of breast cancer cells, but could not affect the proliferation of breast cancer cells. However, Spengler et al. [30] suggested that Excessive clock expression in the nucleus may activate the expression of NF-κB, leading to excessive inflammatory cytokines, while BMAL1 down-regulates this activation. The specific mechanism remains to be further studied.

Lou et al. [39] suggested that BMAL1 might work as a transcriptional factor or transcriptional cofactor which interacted with other transcription factors to regulate the expression of MMPs. Members of the MMP family are zinc-dependent proteinase which can lead to the instability of the extracellular matrix and the deposited non-matrix proteins [40]. MMP9 and MMP2 are common indicators of cancer migration and invasion [41]. JUNG et al. [42] have shown that BMAL1 can weaken the invasion of lung cancer cells by inhibiting the PI3K-Akt-MMP-2 pathway. This result supports the idea that there is a strong molecular link between circadian rhythms and matrix metalloproteinases. However, we did not detect the changes of MMP2 protein in the breast cancer cell. BMAL1 mainly affects the expression of MMP9, which promoting the invasion and metastasis of breast cancer. The results above may result from the differential expression of BMAL1 in various cancer cell lines, so that BMAL1 may regulate the occurrence and development of cancers through different signaling pathways. RT-PCR and western blot showed that overexpression of *Bmal1* could up-regulate the expression of MMP9, and luciferase reporter gene showed that *Bmal1* could promote the promoter activity of MMP9. It is well known that MMP9 is a downstream factor regulated by NF-κB, and NF-κB can promote breast cancer metastasis by promoting the expression of MMP9 [43]. In addition to MMP9, BMAL1 also up-regulated the downstream target genes of NF-κB in breast cancer cells, such as TNF, IL8, uPA, and luciferase reporter gene showed that BMAL1 could activate the activity of NF-κB reporter gene. This suggests that BMAL1 may regulate MMP9 expression through the NF-κB signaling pathway. The NF-κB binding site was present in the MMP9 promoter region. After the deletion or mutation of the NF-κB binding site, the influence of BMAL1 on the MMP9 promoter region was significantly reduced. But we were surprised to find that the activation didn’t disappear entirely. Therefore, there might be other transcription factors affecting the activity of MMP9 promoter. Therefore, NF-κB binding site can affect the regulation of MMP9 expression by BMAL1. When the NF-κB signaling pathway is activated, p65 and p50 form a dimer to bind to the promoter region of the target gene to achieve the regulation of the target gene [44]. Studies have found that some genes can bind to p65 as co-transcription factors, affecting the activity of p65 and thereby promoting or inhibiting the expression of p65 target genes [45]. Therefore, we tested whether the transcription factors NF-κB p50/p65 was involved in the influence of BMAL1 on MMP9 promoter. CoIP assays show that there is an interaction between BMAL1 and p65, while p50 does not. And this interaction was important for the regulation of the NF-κB element of the MMP9 promoter by BMAL1.

Further assays found that overexpression of BMAL1 does not affect the combination of p65 and IκBα. Previous studies have proved that BMAL1 can strengthen p65 through recruiting CBP acetylation and activation of NF-κB [46], the CoIP assay found that BMAL1 can interact with CBP, overexpression of BMAL1 can increase the BMAL1-p65 compound CBP in abundance, as a result, BMAL1 can increase the recruitment of CBP to enhance p65 activity, and further enhance the expression of MMP9 target genes, promote the invasion and metastasis of breast cancer cells.

In a word, our studies showed that BMAL1 could promote the tumor metastasis by increasing the expression of MMP9 at mRNA and protein levels. Further mechanistic details that came to light centered on NF-kB site on the MMP9 promoter that facilitated its up-regulation, providing a new mechanism underlying the invasiveness of breast cancer. Moreover, the expressions of some other NF-kB target genes were also up-regulated by BMAL1, and this may be caused by the accumulation of CBP which promoted the acetylation of p65, recruited by BMAL1 to the NF-kBp65. Induction of BMAL1 expression may provide a strategy for suppression of breast cancer metastasis.

## Conclusions

**Fig. 6:**
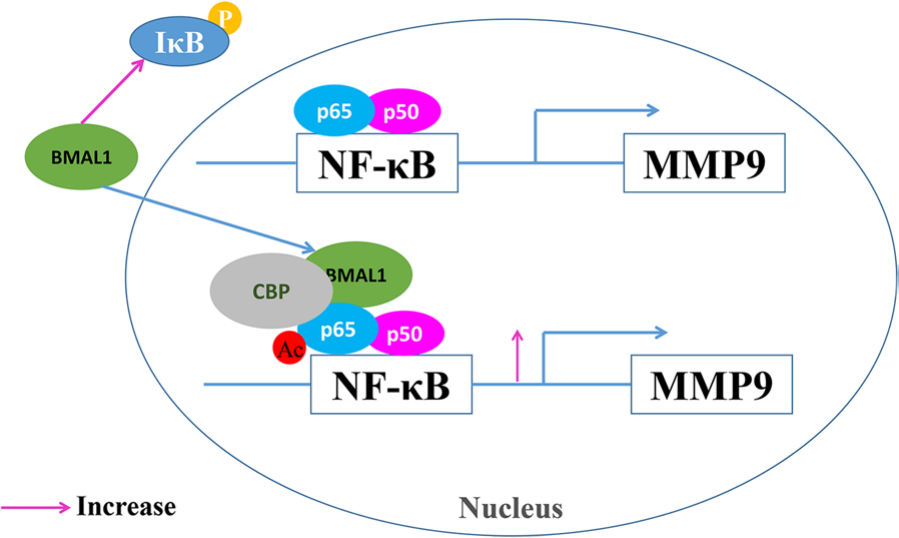
The molecular mechanism by which BMAL1 regulates the expression of MMP9. BMAL1 activated the NF-κB signaling pathway by increasing the phosphorylation of IκB in the cytoplasm. And then in the nucleus BMAL1 recruit CBP to promote the acetylation level of p65, further activating the transcriptional activity of p65, resulting in the up-regulating the expression of MMP9

In this study, we identified that BMAL1 promoted the invasion and metastasis of breast cancer cells through regulating the expression of MMP9, and the possible underlying mechanism is mainly recruiting CBP to increase the acetylation level of p65, further activate the NF-κB signaling pathway (Fig. 6). These data suggest that BMAL1 promotes breast cancer metastasis and can be a candidate as a new prognostic marker and target for the treatment of breast cancer.

## Data Availability

Please contact the corresponding author for all data requests

## Abbreviations

BMAL1: brain and muscle arnt-like 1
CBP: CREB binding protein
CoIP: co-immunoprecipitation
ECM: extracellular matrix
MMP2: matrix metalloproteinase 2
MMP9: matrix metalloproteinase9
SCN: suprachiasmatic nucleus
